# FANSY POSTCOV: A composite clinical immunological predictive index for post‐COVID‐19 syndrome unveils distinctive features in a cohort study of mild to critical patients

**DOI:** 10.1002/ctm2.623

**Published:** 2021-11-24

**Authors:** Jiram Torres‐Ruiz, Julieta Lomelín‐Gascón, Jaquelin Lira‐Luna, Alfredo Pérez‐Fragoso, Roberto Tapia‐Conyer, Miroslava Nuñez‐Aguirre, Beatriz Alcalá‐Carmona, Abdiel Absalón‐Aguilar, José Luis Maravillas‐Montero, Nancy Raquel Mejía‐Domínguez, Carlos Núñez‐Álvarez, Luis Llorente, Sandra Romero‐Ramírez, Victor Andrés Sosa‐Hernández, Rodrigo Cervantes‐Díaz, Guillermo Juárez‐Vega, David Meza‐Sánchez, Marina Rull‐Gabayet, Luis Alberto Martínez‐Juárez, Linda Morales‐Juárez, Lizeth Naomi López‐López, José Adrián Negrete‐Trujillo, Jorge Abelardo Falcón‐Lezama, Rafael Ricardo Valdez‐Vázquez, Héctor Gallardo‐Rincón, Diana Gómez‐Martín

**Affiliations:** ^1^ Department of Immunology and Rheumatology Instituto Nacional de Ciencias Médicas y Nutrición Salvador Zubirán Mexico City Mexico; ^2^ Emergency Medicine Department Instituto Nacional de Ciencias Médicas y Nutrición Salvador Zubirán Mexico City Mexico; ^3^ Departments of Operative and Global Solutions Fundación Carlos Slim Mexico City Mexico; ^4^ Facultad de Medicina, Universidad Nacional Autónoma de México Mexico City Mexico; ^5^ Red de Apoyo a la Investigación Universidad Nacional Autónoma de México e Instituto Nacional de Ciencias Médicas y Nutrición Salvador Zubirán Mexico City Mexico; ^6^ Department of Clinical Research London School of Hygiene and Tropical Medicine London UK; ^7^ Temporary COVID‐19 Hospital Mexico City Mexico; ^8^ Centro Universitario de Ciencias de la Salud (CUCS) Universidad de Guadalajara Guadalajara Mexico

Dear Editor,

The post‐coronavirus disease 2019 (COVID‐19) syndrome encompasses the persistence of symptoms for more than 12 weeks after infection onset.^1^ It causes multiorgan damage and disability, has an unclear pathogenesis and lacks biomarkers.^1^ Our study provides evidence of the clinical and immunological signatures of post‐COVID‐19 syndrome to identify at‐risk patients who may benefit from therapy in a timely manner.

In this observational cohort study, 103 patients with a positive polymerase chan reaction (PCR) test for severe acute respiratory syndrome coronavirus 2 (SARS‐CoV‐2) and who received treatment between August 2020 and February 2021 at the Unidad Temporal, a reference hospital for COVID‐19 in Mexico, were followed up until May 2021 (mean follow‐up, 107.82 days). The primary outcome was the presence of post‐COVID‐19 syndrome 12 weeks after infection onset as diagnosed in patients with fatigue and at least two of the multiorgan symptoms previously described.^1^ Symptoms were assessed using a standardised questionnaire, which was developed after a multidisciplinary group reached a consensus as shown in the Supplementary Material. The systems addressed were respiratory, cardiovascular, neurological, gastrointestinal, musculoskeletal, psychological/psychiatric, ear, nose and throat and dermatological.^1^ All subjects provided written informed consent, and the study was approved by the institutional ethical review committee (Reference: 3341).

On the day the patients sought medical attention or were admitted to the hospital (baseline), and at 1 and 3 months later, a venous blood sample was drawn to characterise T‐ and B‐cell subsets, cytokines, chemokines, serum anti‐cellular antibodies, anti‐SARS‐Cov‐2 immunoglobulin G (IgG) antibodies, neutrophil extracellular traps (NETs) and tripartite motif (TRIM)63 as detailed in the Supplementary Material.

Post‐COVID‐19 syndrome was diagnosed in 46.6% of patients (*n* = 48) and was more frequent in women (*n *= 31, 64.6%). Table S1 shows the clinical features of patients with COVID‐19. The clinical and immunological features of patients at baseline according to the development of post‐COVID‐19 syndrome are shown in Tables S2–S5. Patients classified with post‐COVID‐19 syndrome had an increased serum level of interleukin (IL)‐1β (*p* = .01), elevated proportion of CD24^+^CD38^lo/–^ B cells (*p* = .01), enhanced expression of CD57 in CD8^+^ T cells (*p* = .05), decreased proportion of naïve CD4^+^ T cells (*p* = .01) and lower Th17 cells (*p* = .04). Other biological features at baseline were not associated with post‐COVID‐19 syndrome (Table S6). Univariate analysis for the prediction of post‐COVID‐19 syndrome is shown in Table S7. After multivariate analysis, we constructed a composite predictive index for post‐COVID‐19 syndrome, named **FANSY** POSTCOV as an acronym of the following explanatory variables: **F**emale, percentage of CD24^+^CD38^–/lo^ (**A**typical memory B cells), **N**aïve CD4^+^ T cells, expression of the **S**enescence marker CD57 in CD8^+^ T cells and the number of **SY**mptoms at COVID‐19 diagnosis (Figure 1). The cut‐off point and score of each explanatory variable are shown in Table 1. Using a cut‐off point of ≥7, the FANSY POSTCOV index showed a sensitivity of 0.89 (95% confidence interval (CI) 0.77–0.96), specificity of 0.43 (95% CI 0.30–0.57), positive predictive value of 0.58 (95% CI 0.43–0.81), negative predictive value of 0.82 (95% CI 0.65–0.89), positive likelihood ratio of 1.58 (95% CI 1.23–2.04) and a negative likelihood ratio of 0.23 (95% CI 0.09–0.57). The optimism‐corrected predictive parameters are shown in Table 2. The receiver operator curve and calibration plot for the FANSY POSTCOV index are shown in Figure 2.

**FIGURE 1:**
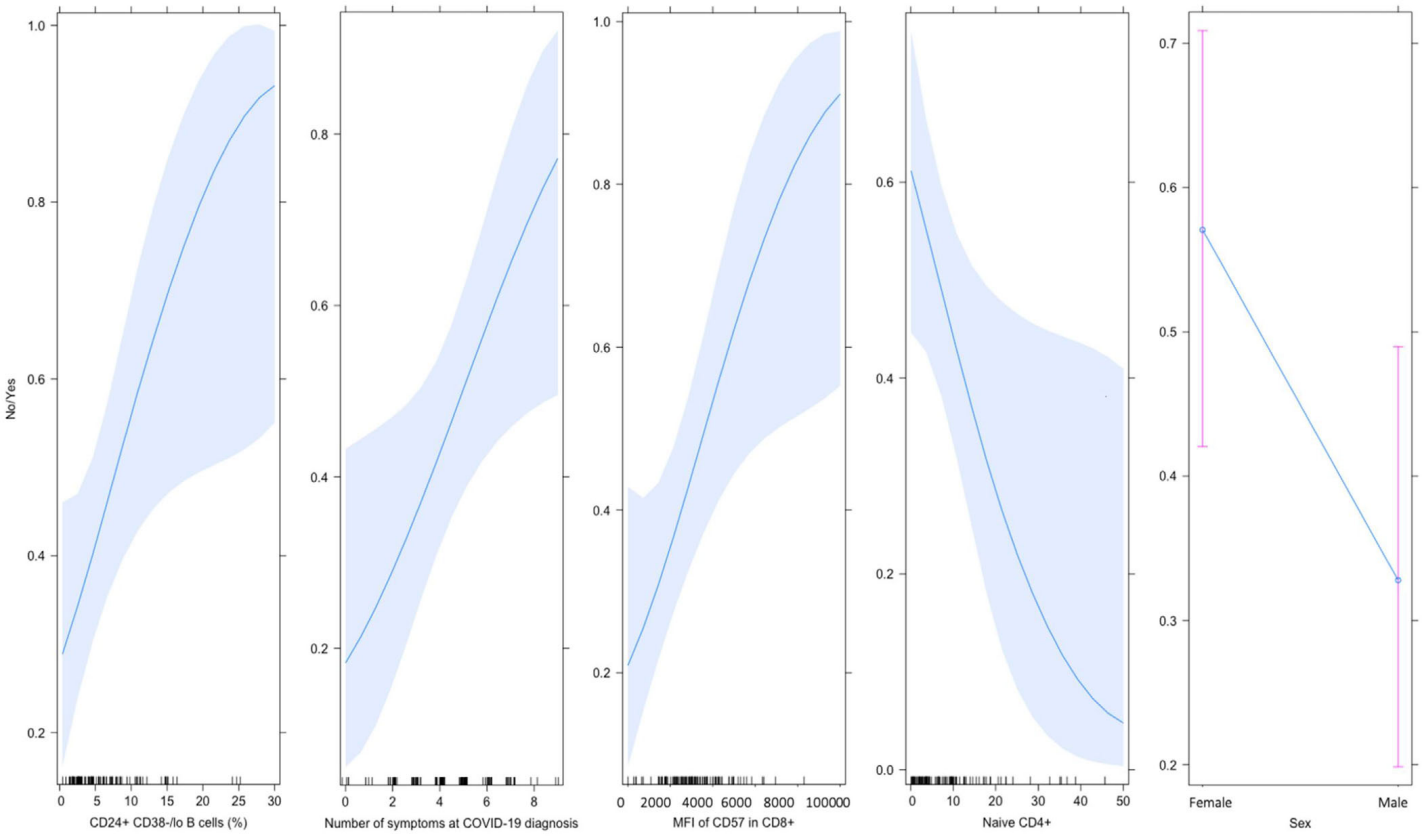
Graphical representation of the effect of the variables included in the FANSY POSTCOV (**F**emale, percentage of CD24^+^CD38^–/lo^ (**A**typical memory B cells), **N**aïve CD4^+^ T cells, expression of the **S**enescence marker CD57 in CD8^+^ T cells and the number of **SY**mptoms at coronavirus disease 2019 (COVID‐19) diagnosis) predictive index for the diagnosis of post‐COVID‐19 syndrome

**TABLE 1:** Post‐COVID‐19 syndrome clinical‐immunological predictive index (FANSY POSTCOV)

	**OR**	**95% CI**	***p*‐value**	**Cut‐off point**	**Score**
Sex	2.73	1.12 to 7.09	0.03	Female	5
Number of symptoms at COVID‐19 diagnosis	1.35	1.06 to 1.77	0.012	>3.5	4
CD24^+^CD38^lo/‐^ B cells	1.13	1.02 to 1.25	0.03	>2.805	3
Naive CD4^+^ T cells	0.93	0.87 to 0.99	0.009	<9.58	2
MFI of CD57 in CD8^+^ T cells	1.00	1.00 to 1.00	0.04	>22,785.5	1

*Note*: All features were evaluated at baseline (at hospital admission).

Abbreviations: COVID‐19, coronavirus disease 2019; CI, confidence interval; FANSY POSTCOV**, F**emale, percentage of CD24^+^CD38^–/lo^ (**A**typical memory B cells), **N**aïve CD4^+^ T cells, expression of the **S**enescence marker CD57 in CD8^+^ T cells and the number of **SY**mptoms at COVID‐19 diagnosis; MFI, mean fluorescence intensity; OR, odds ratio.

**TABLE 2:** Optimism‐corrected predictive parameters of the FANSY POSTCOV composite index

	Original index	Training	Test	Optimism index‐corrected	Optimism
AUC	0.720	0.719	0.720	0.721	−0.001
C‐statistic	0.720	0.719	0.720	0.721	−0.033
Somers’ D	0.440	0.438	0.440	0.443	−0.003
E_max_	0.000	0.000	0.013	0.013	0.013
Brier score	0.213	0.210	0.216	0.220	−0.007

Abbreviations: AUC, area under the curve; FANSY POSTCOV**, F**emale, percentage of CD24^+^CD38^–/lo^ (**A**typical memory B cells), **N**aïve CD4^+^ T cells, expression of the **S**enescence marker CD57 in CD8^+^ T cells and the number of **SY**mptoms at COVID‐19 diagnosis; MFI, mean fluorescence intensity; OR, odds ratio.

**FIGURE 2:**
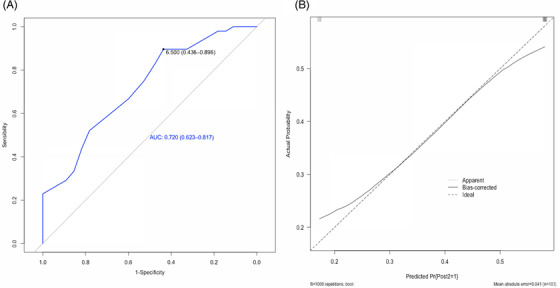
Receiver operator curve and calibration plot for the FANSY POSTCOV index are shown in (A) and (B), respectively; AUC = area under the curve

The serum cytokines, chemokines, anti‐SARS‐CoV‐2 IgG antibodies, NETs and TRIM63 recorded 3 months after COVID‐19 symptom onset or hospital discharge are shown in Table S8. Patients with the post‐COVID‐19 syndrome had higher serum levels of anti‐SARS‐CoV2 IgG antibodies (*p* = .01), granulocyte‐macrophage colony‐stimulating factor (GM‐CSF; *p* = .02) and vascular endothelial growth factor (VEGF; *p* = .009).

Univariate analysis of the biomarkers assessed at post‐COVID‐19 syndrome diagnosis is shown in Table S9. After multivariate analysis, the only variable to remain significant was serum VEGF. The serum VEGF cut‐off point associated with the post‐COVID‐19 syndrome was > 71.71 pg/ml This cut‐off point showed a sensitivity of 0.62 (95% CI 0.48–0.74), specificity of 0.71 (95% CI 0.58–0.81) and positive likelihood ratio of 2.18.

According to previous studies, women and patients with a higher number of symptoms and elevated levels of anti‐SARS‐CoV‐2 antibodies were more prone to post‐COVID‐19 syndrome.^2^ A greater number of symptoms may reflect organ damage, one of the features of post‐COVID‐19 syndrome.^3^ We found that patients with long‐lasting COVID‐19 symptoms showed an exhausted anergic phenotype, with a high proportion of CD24^+^CD38^lo/–^ B cells and elevated expression of CD57 in CD8^+^ T cells, which may promote viral persistence or reactivation.^3^ The CD24^+^CD38^–/lo^ B cell population includes mature naïve and atypical memory B cells, which have been shown to have a dampened proliferative response upon antigenic stimulation.^4^ The naïve CD4+ lymphopenia and the decreased absolute number of Th17 reflect a global defect in the T helper compartment with a reduced viral clearance capacity as was shown in patients with post‐COVID‐19 neurologic symptoms.^5^ A low proportion of naïve CD4+ T cells is also associated with a good prognosis in SARS‐CoV‐2 infection,^6^ and therefore our data may also reflect the survival status of patients with COVID‐19. It has been suggested that post‐COVID‐19 syndrome shares clinical and immunological features with chronic fatigue syndrome and fibromyalgia,^7^ including increased levels of IL‐1β, lower expression of activation markers in T cells and a dysregulated B‐cell compartment.^8^ Our study highlights the serum GM‐CSF as a key driver of COVID‐19 severity^9^ and VEGF as a probable marker of chronic hypoxia after SARS‐CoV‐2‐induced lung damage.^10^

In conclusion, the FANSY POSTCOV index is the first tool to include both clinical and immunological features to predict the development of post‐COVID‐19 syndrome. Our study is also the first to reveal VEGF as a biomarker of this syndrome. We believe that the FANSY POSTCOV will be particularly useful for clinicians to provide closer follow‐up and treatment. Since all our patients are Latin American, additional studies are needed to validate our findings and to elucidate the potential inducers of VEGF in patients with post‐COVID‐19 syndrome. Furthermore, this tool may only be applicable in reference care centres with the capability to measure the included parameters.

## CONFLICT OF INTEREST

All authors had financial support from the Fundación Carlos Slim for the submitted work. There are no financial relationships with any organisations that might have an interest in the submitted work in the previous 3 years and no other relationships or activities that could appear to have influenced the submitted work.

## Supporting information

Supporting Information
