# Supplementary material for: FANSY POSTCOV: A composite clinical immunological predictive index for post‐COVID‐19 syndrome unveils distinctive features in a cohort study of mild to critical patients
Source: Clin Transl Med. 2021 Nov 24;11(11):e623. doi: 10.1002/ctm2.623 (PMC8611773; doi:10.1002/ctm2.623)
Supplement: Supplementary file 1 — Supporting Information [file CTM2-11-e623-s001.docx]

**Supplementary Material**

**Methods**

**Characterization of PBMCs**

Peripheral blood mononuclear cells (PBMCs) were isolated from peripheral blood by density gradient using Ficoll-Paque (GE Healthcare Life Sciences, Chicago, IL, USA). The cells were washed twice with phosphate buffered saline (PBS) and incubated with viability marker Zombie Aqua (Biolegend, San Diego, CA, USA). After two washes with staining buffer (5% fetal bovine serum [FBS] in PBS), the cells were marked for 30 minutes at room temperature (RT) with the following fluorochrome-coupled antibodies: CD19-Pacific Blue, CD3-APC/Fire-750, CD4-Alexa Fluor 488, CD8-PE/Dazzle-594, CD25-BV421, CD45RA-PE/Cy7, CD45RO-PerCP, CD62L-PE, CD127-BV650, CCR7-Alexa Fluor 700, PD-1-APC, CD57-BV785, CD73-BV711, CD21-APC/Fire-750, CD24-PerCP, CD11c-PE/Dazzle-594, IgM-PE, CD27-APC, CD38-Alexa Fluor 488, IgD-Alexa Fluor 700, and CD138-BV605 (all from Biolegend). For the quantification of the T helper and cytotoxic T cell subsets, PBMCs were stimulated with phorbol 12-myristate 13-acetate (50 ng/mL), ionomycin (1 µg/mL), and monensin (4 µL/6 mL) for 5 hours at 37 °C. After fixation and permeabilization using the Cytofix/Cytoperm fixation/permeabilization kit (BD Biosciences, Franklin Lake, NJ, USA), the cells were stained with the following fluorochrome coupled antibodies: IFN-γ-APC, IL-4-PE, and IL-17-BV421 (all from Biolegend). A 4-laser LSR Fortessa flow cytometer (BD Biosciences) was used to acquire the samples.

The following lymphocyte subsets were characterized: CD4^+^ T cells (CD3^+^CD4^+^), CD8^+^ T cells (CD3^+^CD8^+^), naïve T cells (CD3^+^CD4^+^ or CD8^+^CD45RA^+^CD45RO^-^), memory T cells (CD3^+^CD4^+^ or CD8^+^CD45RA^-^CD45RO^+^), central memory T cells (CD3^+^CD4^+^ or CD8^+^CD45RA^-^CD45RO^+^CD62L^+^CCR7^+^), effector memory T cells (CD3^+^CD4^+^ or CD8^+^CD45RA^-^CD45RO^+^CD62L^-^CCR7^-^), exhausted T cells (CD3^+^CD4^+^CD8^+^PD-1^+/hi^), senescent T cells (CD3^+^CD4^+^CD8^+^CD73^+^), anergic T cells (CD3^+^CD4^+^CD8^+^CD57^+^), Th1 (CD4^+^IFN-γ^+^), Th2 (CD4^+^IL-4^+^), Th17 (CD4^+^IL-17^+^), Tc1 (CD8^+^IFNγ^+^), Tc2 (CD8^+^IL-4^+^), Tc17 (CD8^+^IL-17^+^), regulatory T cells (CD4^+^CD25^hi^CD127^lo/-^), total B cells (CD3^-^CD19^+^), CD21^-^ transitional B cells (CD19^+^CD27^-^CD38^hi^CD24^hi^CD21^-/lo^), CD21^+^ transitional B cells (CD19^+^CD27^-^CD38^hi^CD24^lo^CD21^+^), resting naïve B cells (CD19^+^CD27^-^IgD^+^CD38^-^CD24^-^CD11c^-^), activated naïve B cells (CD19^+^CD27^-^IgD^+^CD38^-^CD24^-^CD11c^+^), mature B cells (CD19^+^CD27^-^CD24^-/lo^), plasmablasts (CD19^+^CD27^hi^ CD38^hi^), unswitched classical memory B cells (CD19^+^CD27^+^IgD^+^), switched classical memory B cells (CD19^+^CD27^+^IgD^-^), non-classical CD27^-^IgD^-^ memory B cells (CD19^+^CD38^-/lo^CD24^+^CD27^-^IgD^-^), and non-classical CD27^-^IgD^+^ memory B cells (CD19^+^CD38^-/lo^CD24^+^CD27^-^IgD^+^ ).The absolute number of the B and T subsets were calculates according to the total lymphocyte count in a blood sample drawn the same day. The samples were analyzed using FlowJo v10.7 software (BD Biosciences).

**Assessment of cytokine and chemokine profiles**

The serum concentration of 29 cytokines and chemokines were measured using the MILLIPLEX Multi-Analyte Profiling Human Cytokine/Chemokine Magnetic Bead Panel 29-plex kit (EMD Millipore, Darmstadt, Germany). The following serum cytokines and chemokines were quantified: interleukin 1-α (IL-1α), IL-1β, IL-1RA, IL-2, IL-3, IL-4, IL-5, IL-6, IL-7, IL-8, IL-10, IL-12p40, IL-12p70, IL-13, IL-15, IL-17A, interferon α-2 (IFNα2), IFNγ, tumor necrosis factor-α (TNF-α), TNF-β, monocyte chemoattractant protein 1 (MCP-1)/CCL2, macrophage inflammatory protein 1-α (MIP-1α)/CCL3, macrophage inflammatory protein-1β (MIP-1β)/CCL4, IFNγ-induced protein (IP-10)/CXCL10, eotaxin-1/CCL11, epidermal growth factor (EGF), granulocyte colony-stimulating factor (G-CSF), granulocyte-macrophage colony-stimulating factor (GM-CSF), and vascular endothelial growth factor (VEGF). The samples were acquired with a 2-laser Bio-Plex 200 suspension array system coupled to a Bio-Plex Pro Wash Station (Bio-Rad, Hercules, CA, USA) and analyzed with the Bio-Plex Manager software v6.2 (Bio-Rad).

**Assessment of circulating NETs**

The serum levels of neutrophil elastase (NE)-DNA complexes (NETs) were measured by ELISA as previously described.^1^ Briefly, high-binding 96-well plates were coated with mouse anti-human neutrophil elastase (Calbiochem, Darmstadt, Germany) diluted 1:2000 in coating buffer from the cell death detection ELISA kit (Roche, Basil, Switzerland) and incubated overnight at 4 ºC. After washing three times with PBS/Tween20, the non-specific binding sites were blocked with 1% bovine serum albumin (BSA) for 6 hours at RT. The serum samples were diluted 1:10 in BSA and incubated overnight at 4 ºC. After three washes with PBS/Tween 20, the plates were incubated with the anti-human DNA-POD antibody from the cell death detection ELISA kit (Roche) at a 1:10 dilution in incubation buffer, for 1 hour at RT. The plates were then washed five times with PBS/Tween20 and TMB substrate was added to each well (Thermofisher Scientific, Walthem, MA, USA). The reaction was stopped with stop solution and the plates were read at 450 nm in a the Tecan Sunrise-RC/ ST Evolyzer Plate Reader. We calculated the optic density index as previously described.^1^

**Assessment of the muscle atrophy marker TRIM63 in serum**

Serum levels of TRIM63 were assessed using a TRIM63 ELISA kit according to the manufacturer instructions (MyBioSource, San Diego, CA, USA).

**Assessment of anti-cellular and anti-SARS-CoV-2 IgG antibodies**

Indirect immunofluorescence with the HEp-2 cell line as substrate was used to detect AC/antinuclear IgG antibodies with the NOVA Lite Hep-2 ANA kit according to the manufacturer instructions (INOVA Diagnostics, San Diego, CA, USA). The samples were tested at a 1:40 dilution using a Bee Line System (HTZ, East Grinstead, West Sussex, UK). Three experts read and registered the patterns and titers of AC antibodies using the AutoCyte Image Titer software (Burlington, NC, USA).

Antibodies against the spike S1 domain of SARS-CoV-2 (anti-SARS-CoV-2 IgG) were measured using an anti-SARS-CoV-2 QuantiVac ELISA IgG Kit (EUROIMMUN; Lübeck, Germany) according to manufacturer instructions. A DSX System (DYNEX Technologies, Chantilly, VA, USA) was used to process the samples. We calculated the cut-off value to be <0.51 AU at the 99th percentile using serum samples from our healthy donor bank that were collected from 2017 to 2018. Using this cut-off, we observed a sensitivity of 98.2%, a specificity of 98.9%, and an area under the curve (AUC) of 0.99.

**Statistical analysis**

Since there are no previous studies addressing the effect of the variables that we measured on the development of post-COVID-19 syndrome, we did not calculate a sample size. The comparison among medians was assessed with the Kruskal-Wallis and Dunn’s multiple comparison tests. Imputations were made by a random forest trained algorithm. Missing values were predicted using a random forest trained algorithm.^2^ After discarding highly correlated variables, a binomial logistic regression was used to develop the post-COVID-19 syndrome predictive model. The best-fitting model was selected according to the minimum Akaike information criteria. Assumption of proportionality odds was verified using the Brant and Hosmer-Lemeshow tests.

The internal calibration and performance of the model were assessed using the following parameters: AUC, C-statistic, Spearman's 𝜌, Somers’ D, R^2^, and optimism-corrected overfitting. The calibration plot and discrimination indexes were created by bootstrapping 1000 samples of the original data. The goodness-of-fit of the model was assessed using the Lipsitz test^3^ and the accuracy parameters (sensitivity, specificity, positive predictive value, negative predictive value, positive likelihood ratio, and negative predictive value) were evaluated by cross-validation. The cut-off point for each explanatory variable was calculated using the Youden method, and the AUC and likelihood ratio were calculated using receiving operator curves. A score that was proportional to the magnitude of the odds ratio of each explanatory variable was assigned to construct the predictive index. Statistical analyses were made with the R project software (version 4.0.3). The predictive index was developed and reported according to the TRIPOD statement.^4^

**Supplementary References**

1. Lood C, Blanco LP, Purmalek MM, et al. Neutrophil extracellular traps enriched in oxidized mitochondrial DNA are interferogenic and contribute to lupus-like disease. N*at Med*. 2016;22(2):146-53. doi:10.1038/nm.4027.

2. Stekhoven DJ, Buhlmann P. MissForest--non-parametric missing value imputation for mixed-type data. *Bioinformatics*. 2012;28(1):112-8. doi:10.1093/bioinformatics/btr597.

3. Lipsitz SR, Buoncristiani JF. A robust goodness-of-fit test statistic with application to ordinal regression models. *Stat Med*. 1994;13(2):143-52. doi:10.1002/sim.4780130205.

4. Zamanipoor Najafabadi AH, Ramspek CL, Dekker FW, et al. TRIPOD statement: a preliminary pre-post analysis of reporting and methods of prediction models. *BMJ Open*. 2020;10(9):e041537. doi:10.1136/bmjopen-2020-041537.

**Tables**

**Supplementary table 1.** Clinical features of patients with COVID-19 at diagnosis and during follow-up

|  | **With post-COVID-19 syndrome**  **n (%)**  **N=48** | **Without post-COVID-19 syndrome**  **n (%)**  **N=55** | **P value** |
| --- | --- | --- | --- |
| **At COVID-19 diagnosis** | | | |
| Low/mild disease | 20 (19.4) | 26 (25.2) | 0.55 |
| Severe disease | 13 (12.6) | 17 (16.5) | 0.55 |
| Critical disease | 15 (14.6) | 12 (11.7) | 0.55 |
| Type 2 diabetes mellitus | 8 (7.8) | 16 (15.5) | 0.13 |
| Hypertension | 10 (9.7) | 18 (17.5) | 0.17 |
| Obesity | 21 (20.4) | 25 (24.3) | 0.86 |
| Cardiovascular disease | 0 (0.0) | 3 (2.9) | 0.04 |
| Fever | 28 (27.2) | 31 (30.1) | 0.84 |
| Headache | 31 (30.1) | 37 (35.9) | 0.77 |
| Dysgeusia | 14 (13.6) | 10 (9.7) | 0.18 |
| Conjunctivitis | 5 (4.8) | 2 (1.9) | 0.16 |
| Diarrhea | 8 (7.8) | 7 (6.8) | 0.57 |
| Arthralgia | 30 (29.1) | 27 (26.2) | 0.17 |
| Myalgias | 31 (30.1) | 30 (29.1) | 0.30 |
| Pharyngodynia | 19 (18.4) | 18 (17.5) | 0.46 |
| Rhinorrhea | 12 (11.7) | 9 (8.7) | 0.27 |
| Anosmia | 19 (18.4) | 13 (12.6) | 0.08 |
| Cough | 35 (34.0) | 36 (35.0) | 0.41 |
| **Two months after COVID-19 onset or hospital discharge** | | | |
| Confusion | 4 (3.8) | 0 (0.0) | 0.01 |
| Fatigue | 30 (29.1) | 3 (2.9) | <0.001 |
| Cough | 1 (0.9) | 1 (0.9) | 0.92 |
| Dyspnea | 11 (10.7) | 1 (0.9) | <0.001 |
| Headache | 13 (12.6) | 1 (0.9) | <0.001 |
| Wheezing | 4 (3.8) | 1 (0.9) | 0.11 |
| Fever | 1 (0.9) | 0 (0.0) | 0.21 |
| Joint pain | 7 (6.7) | 0 (0.0) | <0.001 |
| Increased joint size | 2 (1.9) | 1 (0.9) | 0.47 |
| Inability to dress | 0 (0.0) | 0 (0.0) | 0.49 |
| Inability to walk | 0 (0.0) | 0 (0.0) | 0.01 |
| Inability to open jars | 4 (3.8) | 0 (0.0) | 0.02 |
| Chest pain | 6 (5.8) | 0 (0.0) | 0.001 |
| Orthopnea | 1 (0.9) | 0 (0.0) | 0.21 |
| Peripheral edema | 3 (2.9) | 1 (0.9) | 0.23 |
| Myalgia | 11 (10.7) | 1 (0.9) | <0.001 |
| Dermatosis | 0 (0.0) | 1 (0.9) | 0.26 |
| Paresthesia | 10 (9.7) | 0 (0.0) | <0.001 |
| Decreased visual acuity | 8 (7.8) | 0 (0.0) | <0.001 |
| Decreased concentration | 2 (1.9) | 0 (0.0) | 0.07 |
| Memory decline | 6 (5.8) | 2 (1.9) | 0.08 |
| Alopecia | 7 (6.7) | 2 (1.9) | 0.04 |
| Back pain | 1 (0.9) | 0 (0.0) | 0.21 |
| Anosmia | 0 (0.0) | 0 (0.0) | 0.49 |
| Dysgeusia | 0 (0.0) | 0 (0.0) | 0.49 |
| **Three months after COVID-19 onset or hospital discharge** | | | |
| Confusion | 3 (2.9) | 1 (0.9) | 0.23 |
| Fatigue | 27 (26.2) | 3 (2.9) | <0.001 |
| Cough | 0 (0.0) | 0 (0.0) | 0.49 |
| Dyspnea | 10 (9.7) | 2 (1.9) | 0.005 |
| Headache | 14 (13.6) | 4 (3.9) | 0.002 |
| Wheezing | 2 (1.9) | 0 (0.0) | 0.07 |
| Fever | 0 (0.0) | 0 (0.0) | 0.49 |
| Joint pain | 5 (4.9) | 2 (1.9) | 0.16 |
| Increased joint size | 1 (0.9) | 0 (0.0) | 0.21 |
| Inability to dress | 1 (0.9) | 2 (1.9) | 0.21 |
| Inability to walk | 2 (1.9) | 0 (0.0) | 0.07 |
| Inability to open jars | 2 (1.9) | 0 (0.0) | 0.07 |
| Chest pain | 4 (3.8) | 0 (0.0) | 0.01 |
| Orthopnea | 0 (0.0) | 0 (0.0) | 0.49 |
| Peripheral oedema | 1 (0.9) | 1 (0.9) | 0.92 |
| Myalgia | 11 (10.7) | 3 (2.9) | <0.001 |
| Dermatosis | 0 (0.0) | 0 (0.0) | 0.49 |
| Paresthesia | 6 (5.8) | 2 (1.9) | 0.08 |
| Decreased visual acuity | 3 (2.9) | 1 (0.9) | 0.23 |
| Decreased concentration | 3 (2.9) | 0 (0.0) | 0.03 |
| Memory decline | 5 (4.9) | 0 (0.0) | 0.004 |
| Alopecia | 16 (15.5) | 6 (5.8) | 0.005 |
| Back pain | 5 (4.9) | 1 (0.9) | 0.05 |
| Anosmia | 0 (0.0) | 0 (0.0) | 0.49 |
| Dysgeusia | 0 (0.0) | 0 (0.0) | 0.49 |

**Supplementary table 2.** Demographic, clinical, and laboratory characteristics of patients according to diagnosis of post-COVID-19 syndrome

|  | **With post-COVID-19 syndrome**  **N=48** | **Without post-COVID-19 syndrome**  **N=55** | **P value** |
| --- | --- | --- | --- |
| **Demographic characteristics** | | | |
| Female, n (%) | 31 (64.6) | 25 (45.5) | - |
| Male, n (%) | 17 (35.4) | 30 (54.6) | - |
| Age (years) | 50.5 (41.5 to 59.0) | 50.0 (41.0 to 58.0) | - |
| No comorbidities | 1 (1 to 2) | 1 (1 to 2) | 0.19 |
| **Clinical characteristics** | | | |
| Body mass index (kg/m^2^) | 29.0 (26.0 to 32.0) | 29.0 (26.0 to 32.0) | 0.73 |
| Oxygen saturation (SpO_2_ %) | 94.0 (92.0 to 96.0) | 94.0 (92.0 to 96.0) | 0.53 |
| **Laboratory characteristics** | | | |
| Leukocytes (cells/mm^3^) | 6850.00 (4775.00 to 8375.00) | 7200.00 (5300.00 to 8950.00) | 0.27 |
| Total lymphocytes (cells/mm^3^) | 1392.20 (779.50 to 1970.80) | 1145.20 (824.10 to 1663.20) | 0.56 |
| Total lymphocytes (%) | 20.60 (9.45 to 29.68) | 19.70 (10.80 to 33.70) | 0.60 |
| Total neutrophils (cells/mm^3^) | 5496.00 (3350.00 to 7155.00) | 4151.00 (2725.00 to 6950.00) | 0.21 |
| Total neutrophils (%) | 73.25 (60.85 to 86.08) | 71.30 (53.15 to 83.85) | 0.43 |
| Total monocytes (cells/mm^3^) | 380.30 (291.40 to 489.90) | 419.60 (301.10 to 607.60) | 0.35 |
| Total monocytes (%) | 5.65 (4.17 to 8.00) | 7.35 (4.47 to 10.27) | 0.05 |
| Neutrophil/lymphocyte ratio | 3.55 (2.03 to 9.15) | 3.61 (1.58 to 7.89) | 0.55 |
| Hemoglobin (g/dL) | 14.40 (13.15 to 14.90) | 14.50 (13.30 to 15.38) | 0.52 |
| Hematocrit (%) | 41.65 (39.33 to 45.15) | 42.95 (40.15 to 45.62) | 0.45 |
| Platelets (cells/mm^3^) | 286.00 (200.80 to 260.80) | 272.50 (195.80 to 352.50) | 0.69 |
| Glucose (mg/dL) | 122.50 (103.50 to 148.80) | 107.00 (90.50 to 141.00) | 0.15 |
| HbA1c | 6.30 (5.70 to 7.52) | 6.00 (5.45 to 8.12) | 0.54 |
| Alkaline phosphatase (U/L) | 76.50 (62.50 to 83.87) | 73.00 (59.00 to 91.00) | 0.89 |
| Aspartate aminotransferase (U/L) | 28.50 (17.00 to 47.50) | 26.50 (15.00 to 47.25) | 0.73 |
| Albumin (g/dL) | 3.80 (3.63 to 4.15) | 3.95 (3.60 to 4.20) | 0.25 |
| Creatinine (g/dL) | 0.80 (0.60 to 0.92) | 0.70 (0.60 to 1.00) | 0.72 |
| Ultrasensitive C reactive protein ultrasensitive (mg/dL) | 32.00 (2.92 to 96.03) | 18.91 (4.85 to 67.56) | 0.49 |
| Ferritin (ng/dL) | 320.70 (97.30 to 611.00) | 258.20 (129.00 to 565.00) | 0.60 |
| Creatine phosphokinase (U/L) | 38.00 (24.75 to 62.75) | 31.50 (22.00 to 53.25) | 0.46 |
| D Dimer (ng/mL) | 330.00 (210.00 to 480.00) | 265.00 (189.80 to 500.00) | 0.40 |
| PaO_2_/FiO_2_ | 2.55 (1.23 to 3.15) | 2.53 (1.57 to 3.11) | 0.78 |

Results are shown as median (interquartile range) unless otherwise noted.

**Supplementary table 3.** Baseline serum cytokines and chemokines according to diagnosis of post-COVID-19 syndrome

|  | **With post-COVID-19 syndrome**  **median (IQR)**  **N=48** | **Without post-COVID-19 syndrome**  **median (IQR)**  **N=55** | **P value** |
| --- | --- | --- | --- |
| IL-α (pg/mL) | 2.48 (0.55 to 7.68) | 3.24 (0.55 to 11.11) | 0.86 |
| **IL-1β (pg/mL)** | **1.19 (0.80 to 1.84)** | **0.80 (0.52 to 1.28)** | **0.01** |
| IL-1RA (pg/mL) | 23.53 (15.73 to 46.24) | 26.84 (8.80 to 43.20) | 0.54 |
| IL-2 (pg/mL) | 0.65 (0.32 to 1.28) | 0.60 (0.42 to 0.83) | 0.44 |
| IL-3 (pg/mL) | 0.14 (0.13 to 0.16) | 0.14 (0.13 to 0.15) | 0.30 |
| IL-4 (pg/mL) | 2.75 (2.74 to 25.27) | 2.75 (2.74 to 69.03) | 0.94 |
| IL-5 (pg/mL) | 0.93 (0.65 to 1.39) | 0.87 (0.41 to 1.83) | 0.49 |
| IL-6 (pg/mL) | 4.98 (2.03 to 18.22) | 4.98 (1.36 to 20.12) | 0.85 |
| IL-7 (pg/mL) | 12.62 (8.22 to 17.90) | 12.21 (8.12 to 16.35) | 0.85 |
| IL-8 (pg/mL) | 12.73 (8.98 to 19.37) | 13.38 (8.87 to 20.84) | 0.56 |
| IL-10 (pg/mL) | 8.20 (4.81 to 21.59) | 10.29 (3.77 to 26.00) | 0.63 |
| IL-12p70 (pg/mL) | 1.32 (1.32 to 2.11) | 1.32 (1.31 to 1.38) | 0.28 |
| IL-12p40 (pg/mL) | 1.49 (1.49 to 5.05) | 1.49 (1.49 to 1.67) | 0.38 |
| IL-13 (pg/mL) | 0.81 (0.58 to 1.71) | 0.81 (0.81 to 1.93) | 0.66 |
| IL-15 (pg/mL) | 5.37 (3.78 to 7.18) | 5.67 (2.89 to 8.40) | 0.97 |
| IL-17A (pg/mL) | 0.80 (0.19 to 3.61) | 0.80 (0.11 to 3.99) | 0.78 |
| TNF-α (pg/mL) | 25.16 (17.04 to 33.87) | 21.86 (17.13 to 31.14) | 0.49 |
| TNF-β (pg/mL) | 0.68 (0.40 to 2.78) | 0.64 (0.64 to 2.25) | 0.91 |
| G-CSF (pg/mL) | 22.02 (14.68 to 45.21) | 24.82 (14.68 to 54.64) | 0.63 |
| GM-CSF (pg/mL) | 2.66 (2.66 to 9.94) | 2.66 (2.66 to 2.66) | 0.31 |
| VEGF (pg/mL) | 80.16 (38.58 to 185.24) | 74.6 1(23.85 to 160.04) | 0.48 |
| EGF (pg/mL) | 74.75 (49.70 to 123.34) | 90.07 (58.56 to 139.69) | 0.47 |
| IFN-α2 (pg/mL) | 13.79 (13.79 to 13.80) | 13.79 (13.79 to 13.79) | 0.90 |
| IFN-γ (pg/mL) | 5.16 (3.06 to 17.64) | 3.65 (1.83 to 13.56) | 0.23 |
| MCP-1/CCL2 (pg/mL) | 422.00 (282.5 to 624.1) | 425.50 (292.10 to 596.00) | 0.75 |
| MIP-1α /CCL4 (pg/mL) | 1.03 (1.03 to 2.34) | 1.03 (1.03 to 1.03) | 0.05 |
| MIP-1β /CCL3 (pg/mL) | 37.3 4(25.80 to 49.51) | 39.73 (29.64 to 52.15) | 0.30 |
| IP-10/CXCL10 (pg/mL) | 776.9 (413.20 to 1359.9) | 859.50 (477.20 to 2229.40) | 0.39 |
| Eotaxin/CCL11 (pg/mL) | 95.06 (33.91 to 152.78) | 111.82 (79.06 to 144.10) | 0.57 |

EGF=epidermal growth factor; G-CSF=granulocyte colony-stimulating factor; GM-CSF=granulocyte-macrophage colony-stimulating factor; IFN=interferon; IL=interleukin; IP=IFNγ-induced protein; IQR=interquartile range; MCP=monocyte chemoattractant protein; MFI=mean fluorescence intensity; MIP=macrophage inflammatory protein; TNF=tumor necrosis factor; VEGF=vascular endothelial growth factor.

**Supplementary table 4.** Baseline B cell subsets according to diagnosis of post-COVID-19 syndrome

|  | **With post-COVID-19 syndrome**  **median (IQR)**  **N=48** | **Without post-COVID-19 syndrome**  **median (IQR)**  **N=55** | **P value** |
| --- | --- | --- | --- |
| B cells (%) | 9.05 (5.90 to 16.05) | 11.55 (7.07 to 15.62) | 0.42 |
| B cells (cells/µl) | 100.02 (69.73 to 181.55) | 124.48 (65.67 to 211.64) | 0.55 |
| Memory B cells (%) | 13.65 (6.77 to 17.60) | 11.80 (6.28 to 16.43) | 0.73 |
| Memory B cells (cells/µl) | 11.48 (5.62 to 20.44) | 13.05 (6.64 to 21.66) | 0.73 |
| IgD memory B cells (%) | 1.08 (0.55 to 3.61) | 0.93 (0.47 to 2.32) | 0.31 |
| IgD memory B cells (cells/µl) | 1.65 (0.68 to 3.38) | 1.12 (0.48 to 2.64) | 0.42 |
| Pre-switch memory B cells (%) | 1.45 (0.81 to 2.51) | 1.58 (0.78 to 3.29) | 0.53 |
| Pre-switch memory B cells (cells/µl) | 1.77 (0.69 to 3.07) | 2.25 (0.57 to 5.42) | 0.52 |
| Switched classical memory B cells (%) | 3.85 (2.03 to 7.09) | 4.11 (2.23 to 7.23) | 0.75 |
| Switched classical memory B cells (cells/ µl) | 3.89 (1.84 to 8.96) | 4.70 (2.05 to 11.96) | 0.68 |
| Unswitched classical memory B cells (%) | 2.59 (1.53 to 5.29) | 2.94 (1.58 to 4.29) | 0.56 |
| Unswitched classical memory B cells (cells/µl) | 2.83 (1.57 to 6.45) | 2.74 (1.02 to 7.95) | 0.61 |
| Plasmablasts (%) | 1.85 (1.19 to 4.81) | 2.28 (1.05 to 4.22) | 0.78 |
| Plasmablasts (cells/µl) | 2.28 (1.16 to 5.32) | 2.47 (1.28 to 6.16) | 0.86 |
| Plasma B cells (%) | 0.45 (0.21 to 0.75) | 0.37 (0.22 to 0.70) | 0.67 |
| Plasma B cells (cells/µl) | 0.53 (0.26 to 1.14) | 0.41 (0.24 to 1.00) | 0.70 |
| CD138^-^ plasma B cells (%) | 1.34 (0.51 to 3.64) | 1.63 (0.69 to 3.22) | 1.00 |
| CD138^-^ plasma B cells (cells/µl) | 1.65 (0.64 to 3.62) | 1.71 (0.61 to 4.57) | 0.80 |
| CD27^-^ B cells (%) | 81.70 (74.90 to 88.05) | 82.40 (77.33 to 89.60) | 0.45 |
| CD27^-^ B cells (cells/µl) | 81.46 (54.52 to 134.62) | 106.16 (49.08 to 170.53) | 0.47 |
| T1^+^T2 B cells (%) | 6.41 (4.93 to 8.62) | 7.54 (5.47 to 11.90) | 0.18 |
| T1^+^T2 B cells (cells/µl) | 8.03 (5.04 to 11.31) | 10.32 (5.62 to 19.18) | 0.25 |
| Transitional CD21^-^ B cells (%) | 1.45 (0.86 to 3.00) | 2.30 (1.27 to 5.66) | 0.06 |
| Transitional CD21^-^ B cells (cells/µl) | 1.78 (0.94 to 3.14) | 2.78 (1.23 to 7.26) | 0.09 |
| Transitional CD21^+^ B cells (%) | 4.24 (3.11 to 5.57) | 3.88 (2.98 to 5.15) | 0.63 |
| Transitional CD21^+^ B cells (cells/µl) | 4.44 (2.70 to 6.95) | 4.86 (2.67 to 9.56) | 0.57 |
| **CD24^+^CD38^lo/-^ B cells (%)** | **6.21 (3.65 to 10.82)** | **4.05 (2.75 to 6.73)** | **0.01** |
| CD24^+^CD38^lo/-^ B cells (cells/µl) | 8.74 (3.96 to 12.53) | 4.62 (2.53 to 10.86) | 0.11 |
| IgD^-^ B cells (%) | 1.18 (0.55 to 2.46) | 0.89 (0.53 to 1.72) | 0.20 |
| IgD^-^ B cells (cells/µl) | 1.47 (0.60 to 3.71) | 1.27 (0.47 to 2.99) | 0.36 |
| **IgD^+^ B cells (%)** | **2.90 (1.30 to 4.49)** | **1.80 (1.21 to 2.91)** | **0.04** |
| IgD^+^ B cells (cells/µl) | 3.06 (1.74 to 7.04) | 2.12 (1.09 to 4.17) | 0.06 |
| Mature B cells (%) | 61.20 (51.42 to 70.83) | 66.25 (55.08 to 64.90) | 0.07 |
| Mature B cells (cells/µl) | 63.25 (39.31 to 104 to 17) | 82.04 (37.30 to 146.81) | 0.37 |
| Naive B cells (%) | 40.50 (33.52 to 46.70) | 41.65 (35.80 to 48.27) | 0.62 |
| Naive B cells (cells/µl) | 39.70 (27.71 to 74.81) | 54.57 (23.34 to 89.00) | 0.67 |
| Resting naive B cells (%) | 3.74 (2.20 to 27.35) | 3.86 (2.82 to 9.07) | 0.83 |
| Resting naive B cells (cells/µl) | 6.27 (3.42 to 18.50) | 5.91 (2.48 to 14.80) | 0.42 |
| Activated naive B cells (%) | 32.30 (4.67 to 40.40) | 33.50 (17.07 to 43.08) | 0.27 |
| Activated naive B cells (cells/µl) | 34.02 (3.96 to 59.16) | 35.36 (13.28 to 68.32) | 0.42 |

EGF=epidermal growth factor; G-CSF=granulocyte colony-stimulating factor; GM-CSF=granulocyte-macrophage colony-stimulating factor; IFN=interferon; IL=interleukin; IP=IFNγ-induced protein; IQR=interquartile range; MCP=monocyte chemoattractant protein; MFI=mean fluorescence intensity; MIP=macrophage inflammatory protein; TNF=tumor necrosis factor; VEGF=vascular endothelial growth factor.

**Supplementary table 5.** Baseline T cell subsets according to diagnosis of post-COVID-19 syndrome

|  | **With post-COVID-19 syndrome**  **median (IQR)**  **N=48** | **Without post-COVID-19 syndrome**  **median (IQR)**  **N=55** | **P value** |
| --- | --- | --- | --- |
| Absolute lymphocytes | 1339.80 (769.10 to 1957.00) | 1145.20 (824.10 to 1663.20) | 0.56 |
| **CD4^+^ T cells (%)** | **14.65 (9.45 to 20.70)** | **21.80 (12.05.31.88)** | **0.02** |
| T CD4^+^ T cells (cells/µl) | 197.25 (81.65 to 369.44) | 233.40 (129.68 to 377.05) | 0.23 |
| CD4^+^CD57^+^ T cells (%) | 0.71 (0.29 to 1.62) | 0.88 (0.23 to 1.30) | 0.94 |
| CD4^+^CD57^+^ T cells (cells/µl) | 1.44 (0.43 to 3.15) | 2.04 (0.24 to 4.76) | 0.28 |
| MFI of CD57 in CD4^+^ T cells | 27019 (13668 to 36774) | 20948 (8848 to 34370) | 0.20 |
| CD4^+^CD73^+^ T cells (%) | 1.53 (0.82 to 2.54) | 2.06 (0.94 to 2.98) | 0.16 |
| CD4^+^CD73^+^ T cells (cells/µl) | 2.51 (0.91 to 6.77) | 4.73 (1.62 to 10.68) | 0.12 |
| MFI of CD73 in CD4^+^ T cells | 3014 (2649 to 3506) | 3290 (2737 to 3823) | 0.32 |
| CD4^+^PD-1^+^ T cells (%) | 2.05 (0.93 to 3.39) | 2.32 (1.48 to 3.39) | 0.51 |
| CD4^+^PD-1^+^ T cells (cells/µl) | 3.75 (0.90 to 9.81) | 5.64 (2.76 to 12.26) | 0.19 |
| MFI of PD-1 in CD4^+^ T cells | 3336 (2074 to 2595) | 2368 (2055 to 2639) | 0.86 |
| Regulatory T cells (%) | 0.33 (0.09 to 0.78) | 0.32 (0.19 to 0.65) | 0.77 |
| Regulatory T cells (cells/µl) | 4.25 (0.67 to 9.12) | 4.12 (1.73 to 8.11) | 0.81 |
| Memory CD4^+^ T cells (%) | 7.00 (4.56 to 10.97) | 8.16 (5.20 to 0.05) | 0.65 |
| Memory CD4^+^ T cells (cells/µl) | 12.80 (4.33 to 36.08) | 18.03 (7.15 to 45.71) | 0.45 |
| Effector memory CD4^+^ T cell (%) | 2.21 (1.45 to 3.36) | 2.16 (1.32 to 3.31) | 0.81 |
| Effector memory CD4^+^ T cells (cells/µl) | 4.58 (1.69 to 7.77) | 4.59 (2.48 to 13.75) | 0.38 |
| Central memory CD4^+^ T cells (%) | 2.29 (0.72 to 4.67) | 2.92 (1.15 to 5.65) | 0.49 |
| Central memory CD4^+^ T cells (cells/µl) | 3.66 (0.67.17.10) | 6.19 (1.67 to 20.23) | 0.25 |
| **Naive CD4^+^ T cells (%)** | **4.37 (2.04 to 9.28)** | **7.50 (3.61 to 15.82)** | **0.01** |
| **Naive CD4^+^ T cells (cells/µl)** | **8.93 (1.34 to 21.50)** | **20.23 (4.08 to 55.35)** | **0.04** |
| CD8^+^ T cells (%) | 8.74 (5.95 to 11.85) | 11.10 (5.99 to 15.43) | 0.29 |
| CD8^+^ T cells (cells/µl) | 100.27 (59.52 to 208.7) | 128.79 (52.17 to 233.47) | 0.91 |
| CD8^+^CD57^+^ T cells (%) | 2.52 (1.59 to 5.04) | 2.96 (1.03 to 6.42) | 0.93 |
| CD8^+^CD57^+^ T cells (cells/µl) | 3.55 (0.98 to 11.03) | 4.17 (0.71 to 10.92) | 0.86 |
| **MFI of CD57 on CD8^+^ T cells** | **30986 (24150 to 41965)** | **27570 (17875 to 37245)** | **0.05** |
| CD8^+^PD-1^+^ T cells (%) | 1.97 (1.05 to 3.57) | 1.84 (0.92 to 3.61) | 0.68 |
| CD8^+^PD-1^+^ T cells (cells/µl) | 2.75 (0.65 to 5.68) | 3.16 (0.53 to 5.70) | 0.98 |
| MFI of PD-1 in CD8^+^ T cells | 2010 (1803 to 2488) | 2010 (1739 to 2330) | 0.48 |
| CD8^+^CD73^+^ T cells (%) | 1.77 (1.11 to 3.15) | 2.83 (1.18 to 4.28) | 0.14 |
| CD8^+^CD73^+^ T cells (cells/µl) | 1.62 (0.79 to 5.31) | 2.66 (0.82 to 10.91) | 0.32 |
| MFI of CD73 in CD8^+^ T cells | 3875 (3309 to 4368) | 3998 (3276 to 4719) | 0.97 |
| Memory CD8^+^ T cells (%) | 0.43 (0.17 to 0.80) | 0.23 (0.12 to 0.58) | 0.09 |
| Memory CD8^+^ T cells (cells/µl) | 0.44 (0.14 to 1.23) | 0.28 (0.07 to 1.03) | 0.36 |
| Effector memory CD8^+^ T cells (%) | 0.26 (0.09 to 0.50) | 0.15 (0.07 to 0.41) | 0.33 |
| Effector memory CD8^+^ T cells (cells/µl) | 0.27 (0.06 to 0.84) | 0.21 (0.03 to 0.94) | 0.65 |
| Central memory CD8^+^ T cells (%) | 0.01 (0.00 to 0.04) | 0.00 (0.00 to 0.03) | 0.40 |
| Central memory CD8^+^ T cells (cells/µl) | 0.00 (0.00 to 0.05) | 0.00 (0.00 to 0.05) | 0.65 |
| Naive CD8^+^ T cells (%) | 5.45 (3.98 to 7.00) | 7.01 (3.74 to 10.82) | 0.32 |
| Naive CD8^+^ T cells (cells/µl) | 4.91 (2.21 to 13.77) | 9.02 (2.07 to 22.57) | 0.51 |
| Th1 (%) | 29.05 (20.35 to 39.60) | 31.41 (21.95 to 37.95) | 0.76 |
| Th1 (cells/µl) | 42.65 (19.21 to 84.84) | 48.67 (30.55 to 117.45) | 0.28 |
| Th2 (%) | 15.15 (9.39 to 26.35) | 18.80 (13.03 to 29.45) | 0.12 |
| Th2 (cells/µl) | 26.62 (11.92 to 55.50) | 35.75 (22.29 to 53.14) | 0.08 |
| Th17 (%) | 0.33 (0.17 to 2.04) | 0.70 (0.21 to 2.08) | 0.50 |
| **Th17 (cells/µl)** | **0.61 (0.30 to 2.28)** | **1.64 (0.56 to 4.07)** | **0.04** |
| Tc1 (%) | 35.10 (24.00 to 48.85) | 37.25 (29.25 to 47.95) | 0.71 |
| Tc1 (cells/µl) | 44.65 (19.28 to 98.71) | 66.27 (25.24 to 102.40) | 0.39 |
| Tc2 (%) | 19.85 (13.30 to 37.52) | 18.30 (14.43 to 25.77) | 0.96 |
| Tc2 (cells/µl) | 24.03 (12.10 to 62.74) | 35.17 (17.13 to 57.24) | 0.34 |
| Tc17 (%) | 0.09 (0.03 to 0.51) | 0.14 (0.05 to 0.54) | 0.48 |
| Tc17 (cells/µl) | 0.16 (0.05 to 0.85) | 0.27 (0.10 to 0.81) | 0.30 |

IQR=interquartile range; MFI=mean fluorescence intensity.

**Supplementary table 6.** Serum levels of anti-SARS-CoV-2 immunoglobulin G antibodies, NETs, and TRIM63 according to diagnosis of post-COVID-19 syndrome

|  | **With post-COVID-19 syndrome**  **median (IQR)**  **N=48** | **Without post-COVID-19 syndrome**  **median (IQR)**  **N=55** | **P value** |
| --- | --- | --- | --- |
| Anti-SARS-CoV-2 IgG antibodies |  |  |  |
| Baseline (AU) | 5.54 (0.27 to 7.86) | 1.31 (0.19 to 7.03) | 0.12 |
| One month after symptom onset/hospital discharge (AU) | 8.58 (7.68 to 9.40) | 7.84 (6.11 to 8.90) | 0.08 |
| Baseline NETs (AU) | 1.22 (1.00 to 1.51) | 1.31 (1.09to 1.63) | 0.22 |
| Baseline TRIM63 (pg/mL) | 60.13 (24.42 to 235.71) | 73.58 (20.40 to 143.47) | 0.94 |

IgG, immunoglobulin G; IQR=interquartile range; NET=neutrophil extracellular trap; TRIM63=Tripartite motif-containing protein 63.

**Supplementary table 7.** Univariate analysis of assessed baseline parameters used for the prediction of post-COVID-19 syndrome

|  | **P value** | **AUC** | **Sensitivity** | **Specificity** |
| --- | --- | --- | --- | --- |
| Number of symptoms at COVID-19 diagnosis | 0.05 | 0.60 (0.49 to 0.71) | 0.79 | 0.38 |
| Total monocytes (cells/mm^3^) | 0.05 | - | - | - |
| Sex | 0.05 | 0.59 (0.54 to 0.64) | 0.65 | 0.55 |
| Mature B cells (%) | 0.05 | - | - | - |
| IgD^+^ B cells (%) | 0.02 | - | - | - |
| CD24^+^CD38 ^lo/-^ B cells (%) | 0.007 | 0.63 (0.52 to 0.74) | 0.69 | 0.58 |
| Naive CD4^+^ T cells (%) | 0.012 | 0.63 (0.52 to 0.74) | 0.79 | 0.44 |
| Monocytes (%) | 0.05 | - | - | - |
| MFI of PD-1 in CD8^+^ T cells | 0.08 | - | - | - |
| MFI of CD57 in CD8^+^ T cells | 0.08 | 0.60 (0.49 to 0.71) | 0.81 | 0.38 |
| CD73^+^ CD4^+^ T cells (%) | 0.09 | - | - | - |
| CD4^+^ T cells (%) | 0.05 | - | - | - |
| GM-CSF (pg/mL) | 0.10 | - | - | - |
| IL-12p40 (pg/mL) | 0.08 | - | - | - |
| PaO_2_/FiO_2_ | 0.81 | - | - | - |

AUC=area under the curve; MFI=mean fluorescence intensity.

Only the AUC, sensitivity, and specificity of the variables used to construct the index are shown.

**Supplementary table 8.** Levels of serum cytokines, chemokines, anti-SARS-CoV-2 immunoglobulin G antibodies, NETs, and TRIM63 at 3 months follow-up according to diagnosis of post-COVID-19 syndrome

|  | **With post-COVID-19 syndrome**  **median (IQR)**  **N=48** | **Without post-COVID-19 syndrome**  **median (IQR)**  **N=55** | **P value** |
| --- | --- | --- | --- |
| Serum cytokines/chemokines |  |  |  |
| IL-α (pg/mL) | 1.91 (1.91 to 3.89) | 1.91 (1.91 to 5.67) | 0.57 |
| IL-1β (pg/mL) | 1.21 (1.00 to 1.69) | 1.21 (0.96 to 1.52) | 0.14 |
| IL-1RA (pg/mL) | 17.08 (11.15 to 27.43) | 15.62 (10.57 to 24.52) | 0.11 |
| IL-2 (pg/mL) | 1.06 (0.77 to 1.47) | 1.01 (0.74 to 1.32) | 0.28 |
| IL-3 (pg/mL) | 0.30 (0.27 to 0.58) | 0.29 (0.26 to 0.34) | 0.06 |
| IL-4 (pg/mL) | 14.75 (4.00 to 74.73) | 4.00 (4.00 to 64.40) | 0.05 |
| IL-5 (pg/mL) | 1.25 (0.95 to 1.95) | 1.29 (0.95 to 2.03) | 0.67 |
| IL-6 (pg/mL) | 1.72 (1.25 to 3.69) | 1.65 (1.16 to 3.54) | 0.31 |
| IL-7 (pg/mL) | 7.61 (4.71 to 12.02) | 7.42 (4.64 to 10.17) | 0.59 |
| IL-8 (pg/mL) | 6.32 (4.27 to 9.01) | 6.50 (4.35 to 10.19) | 0.72 |
| IL-10 (pg/mL) | 3.16 (2.11 to 4.41) | 3.07 (2.11 to 10.18) | 0.76 |
| IL-12p70 (pg/mL) | 1.79 (1.10 to 2.69) | 1.65 (1.10 to 2.77) | 0.20 |
| IL-12p40 (pg/mL) | 0.27 (0.27 to 6.15) | 0.27 (0.27 to 3.04) | 0.16 |
| IL-13 (pg/mL) | 1.15 (0.62 to 3.98) | 1.15 (0.62 to 4.15) | 0.93 |
| IL-15 (pg/mL) | 1.89 (1.39 to 2.75) | 1.74 (1.34 to 2.42) | 0.21 |
| IL-17A (pg/mL) | 2.54 (1.70 to 4.11) | 2.36 (1.70 to 3.92) | 0.43 |
| TNF-α (pg/mL) | 12.69 (9.77 to 16.23) | 11.84 (9.44 to 15.96) | 0.54 |
| TNF-β (pg/mL) | 1.82 (0.82 to 4.62) | 1.53 (0.82 to 4.66) | 0.61 |
| G-CSF (pg/mL) | 12.35 (4.71 to 23.46) | 10.48 (4.89 to 20.57) | 0.43 |
| **GM-CSF (pg/mL)** | **1.29 (0.73 to 2.37)** | **1.01 (0.66 to 1.80)** | **0.02** |
| **VEGF (pg/mL)** | **65.50 (40.67 to 95.91)** | **58.03 (36.62 to 83.90)** | **0.009** |
| EGF (pg/mL) | 57.71 (31.41 to 131.07) | 52.53 (30.77 to 116.38) | 0.35 |
| IFN-α2 (pg/mL) | 4.22 (4.22 to 4.22) | 4.22 (4.22 to 4.22) | 0.13 |
| IFN-γ (pg/mL) | 5.19 (2.31 to 10.36) | 4.60 (2.08 to 10.06) | 0.21 |
| MCP-1/CCL2 (pg/mL) | 381.20 (282.90 to 493.20) | 377.00 (263.30 to 492.50) | 0.76 |
| MIP-1α /CCL4 (pg/mL) | 2.42 (2.24 to 2.42) | 2.42 (2.42 to 2.42) | 0.13 |
| MIP-1β /CCL3 (pg/mL) | 25.15 (16.50 to 32.82) | 26.78 (19.05 to 33.17) | 0.13 |
| IP-10/CXCL10 (pg/mL) | 317.77 (246.99 to 438.83) | 305.97 (236.65 to 429.26) | 0.50 |
| Eotaxin/CCL11 (pg/mL) | 86.14 (59.60 to 122.37) | 82.49 (58.36 to 137.88) | 0.92 |
| **Anti-SARS-CoV-2 IgG antibodies (AU)** | **8.28 (6.11 to 8.87)** | **6.97 (5.40 to 8.41)** | **0.01** |
| NETs (AU) | 1.00 (0.91 to 1.33) | 0.93 (0.81 to 1.17) | 0.07 |
| TRIM63 (pg/mL) | 0.15 (0.12 to 0.37) | 0.15 (0.11 to 0.36) | 0.72 |

EGF=epidermal growth factor; G-CSF=granulocyte colony-stimulating factor; GM-CSF=granulocyte-macrophage colony-stimulating factor; IFN=interferon; IgG, immunoglobulin G; IL=interleukin; IP=IFNγ-induced protein; IQR=interquartile range; MCP=monocyte chemoattractant protein; MIP=macrophage inflammatory protein; NET=neutrophil extracellular trap; TNF=tumor necrosis factor; TRIM63=Tripartite motif-containing protein 63; VEGF=vascular endothelial growth factor.

**Supplementary table 9.** Univariate analysis of the biomarkers assessed at post-COVID-19 syndrome diagnosis

|  | **OR** | **95% CI** | **P value** |
| --- | --- | --- | --- |
| IFN-α2 | 1.07 | (1.00 to 1.19) | 0.04 |
| VEGF | 1.01 | (1.00 to 1.02) | 0.010 |
| MIP-1α /CCL4 | 2.54 | (0.00 to NA) | 0.08 |
| NETs | 1.24 | (0.46 to 3.69) | 0.66 |
| TRIM63 | 0.88 | (0.08 to 8.92) | 0.92 |
| Anti-SARS-CoV-2 IgG antibodies | 1.14 | (0.97 to 1.35) | 0.11 |

CI=confidence interval; IFN=interferon; IgG, immunoglobulin G; MIP=macrophage inflammatory protein; NET=neutrophil extracellular trap; OR=odds ratio; TRIM63=Tripartite motif-containing protein 63; VEGF=vascular endothelial growth factor.
